# The Rare RNA Methylations m²G, Cm, m⁵U and ms²i⁶A: Roles in Disease Pathogenesis and Emerging Therapeutic Implications

**DOI:** 10.7150/ijms.125197

**Published:** 2026-04-23

**Authors:** Keyu Wan, Tiantian Nie, Qin Zhang, Ying Huang, Jing Bian, Jia Min

**Affiliations:** 1 Department of Anesthesiology, The First Affiliated Hospital of Nanchang University, Nanchang, China.; 2 The First Clinical Medical College, Nanchang University, Nanchang, China.; 3 Department of Oncology, The First Affiliated Hospital of Nanchang University, Nanchang, China.

**Keywords:** RNA modification, N2-methylguanosine (m^2^G), 2′-O-methylcytidine (Cm), 5-methyluridine (m^5^U), 2-methylthio-N6-isopentenyladenosine (ms^2^i^6^A), detection methods, diseases

## Abstract

RNA methylation modifications play a central and multifaceted role in various physiological processes by precisely regulating key steps in the RNA life cycle, including nuclear processing, nuclear export, splicing, and cytoplasmic translation. These modifications, which occur on the four nucleotides that constitute RNA strands, are tightly regulated by specific proteins known as “writers,” “readers,” and “erasers.” Advances in high-throughput sequencing and mass spectrometry technologies have progressively unveiled the biological functions of common RNA methylation marks such as N6-methyladenosine (m^6^A), N1-methyladenosine (m^1^A), and 5-methylcytosine (m^5^C). However, our understanding of how RNA modifications influence various cellular processes remains limited, and research focusing on the biological significance of rare RNA methylation modifications is particularly scarce.

This review shifts the research focus toward several relatively understudied and less widely recognized RNA methylation modifications, providing an in-depth analysis of four specific modifications: N2-methylguanosine (m^2^G), 2′-O-methylcytidine (Cm), 5-methyluridine (m^5^U), and 2-methylthio-N6-isopentenyladenosine (ms^2^i^6^A). It comprehensively elucidates their molecular mechanisms, biological functions, and associations with disease. In addition, this article summarizes the current methodologies available for detecting RNA modifications and discusses the potential applications of these RNA methylation modifications in disease therapy.

## Introduction

RNA methylation is a common post-transcriptional regulatory mechanism, referring to the transfer of a methyl group from a methyl donor to the nucleotide molecules of RNA under the catalysis of RNA methyltransferases 1-3. As early as the 1960s, researchers had observed that specific RNA methyltransferases could alter the structure of tRNA 4. With the continuous advancement of sequencing technologies, the efficiency and accuracy of nucleic acid sequencing have significantly improved. Consequently, in addition to the abundant methylation modifications identified in tRNA and rRNA, diverse types of methylation modifications have also been discovered in many low-abundance RNAs 5-9. In eukaryotic RNAs, common internal methylation modifications include N6-methyladenosine (m^6^A), 5-methylcytosine (m^5^C), 7-methylguanosine (m^7^G), and N1-methyladenosine (m^1^A). RNA methylation can occur on any nucleotide residue, including adenosine, uridine, guanosine, or cytidine, and its regulation requires the coordinated actions of methyltransferases (“writers”), demethylases (“erasers”), and RNA-binding proteins (“readers”) 10,11.

Currently, most studies have primarily focused on well-characterized RNA modifications such as m6A, whereas systematic summaries of other less-studied RNA methylation modifications with important biological functions remain limited. Recent studies have demonstrated that RNA modifications such as N2-methylguanosine (m^2^G), 2′-O-methylcytidine (Cm), 5-methyluridine (m^5^U), and 2-methylthio-N6-isopentenyladenosine (ms^2^i^6^A) play indispensable roles in RNA biosynthesis. N2-methylguanosine is a methylation modification that occurs at the second carbon atom of the ribose moiety in RNA guanosine under the action of methyltransferases. 2′-O-methylcytidine results from the methylation of the 2′-hydroxyl group of the ribose in cytidine, catalyzed by RNA methyltransferases. 5-methyluridine is generated by the methylation of the fifth carbon atom of uridine, catalyzed by pyrimidine methyltransferases. ms^2^i^6^A is a complex modification occurring at position 37 of tRNA, involving the sequential addition of an isopentenyl group at the N6 position and a methylthio. These four modifications are predominantly found in tRNAs, and both m^5^U and m^2^G share the same type of methyltransferase and function through similar structural folding mechanisms 12,13.

In this review, we focus on the structures, biological functions, and disease relevance of four RNA methylation modifications: m^2^G, Cm, m^5^U, and ms^2^i^6^A. Based on recent advances, we summarize the regulatory mechanisms, physiological roles, and associations with diseases of these modifications. We also briefly introduce established and emerging techniques for detecting RNA modification sites. Although RNA modifications have garnered increasing attention in recent years, many mechanistic aspects remain to be elucidated. Therefore, a systematic summary of these relatively understudied RNA modifications will help further elucidate the regulatory network of RNA modifications and provide new perspectives for understanding disease mechanisms and developing potential therapeutic strategies. Accordingly, we also provide a preliminary outlook on future research directions in this field.

## Regulatory Roles of RNA Methylation Modifications

**Figure 1:**
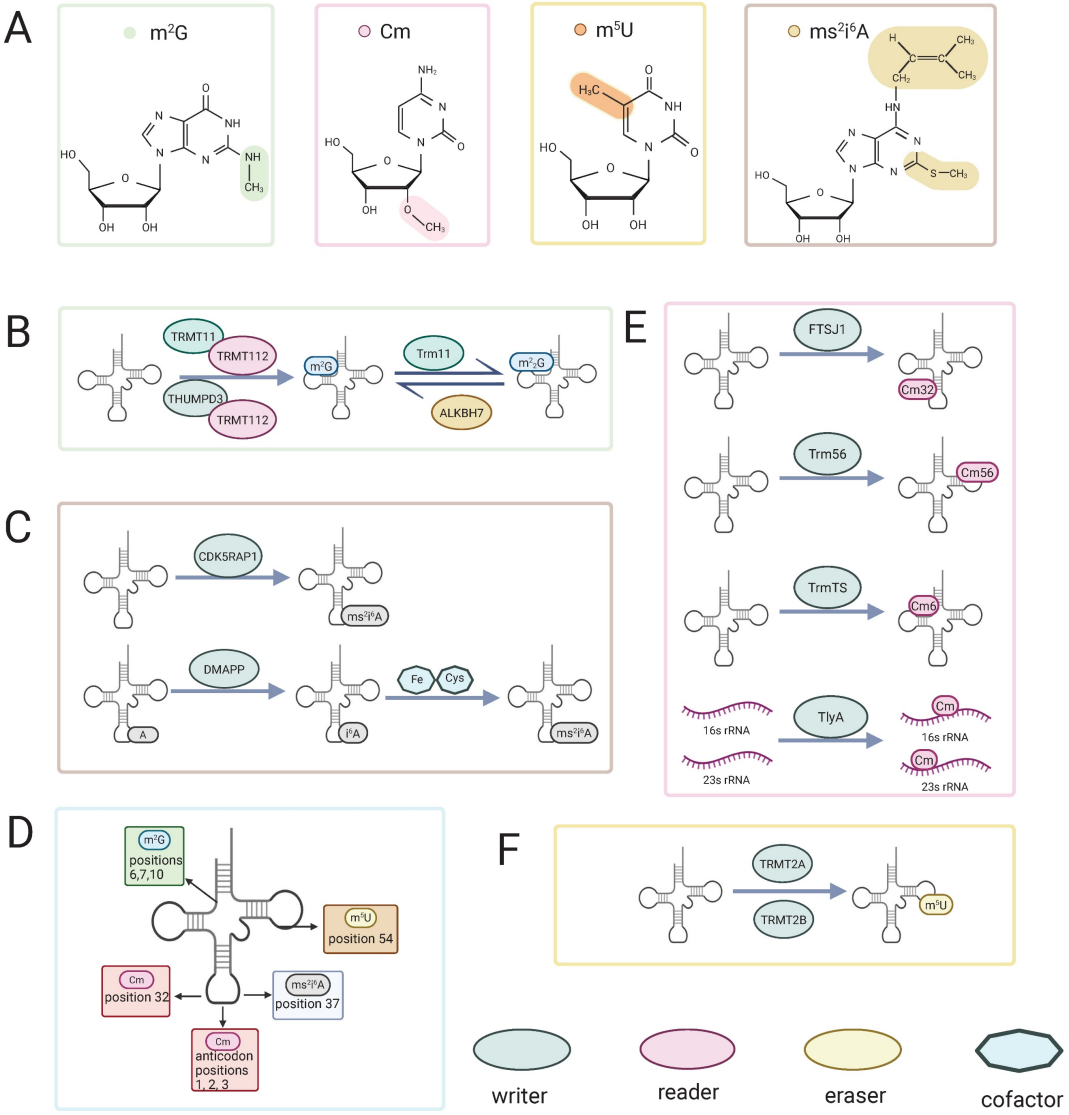
Chemical structures, regulatory enzymes, and tRNA distribution of four rare RNA methylation modifications. (A) Chemical structures of m²G, Cm, m⁵U, and ms²i⁶A.(B, C, E, F) Writers, readers, erasers and cofactors involved in m²G (B), ms²i⁶A (C), Cm (E), and m⁵U (F) methylation. (D) Representative positions of the four modifications on tRNA. Colored shapes denote writers, readers, erasers, and cofactors.

RNA methylation modifications are likely to exert significant effects on various molecular processes, including RNA metabolism, splicing, stability, and translation 14. These modifications can alter the biological properties of RNA, affecting its secondary structure, base-pairing capacity, and interactions with proteins. Notably, modifications within ribosomal RNA (rRNA) have been implicated in ribosome biogenesis 15, while those occurring in transfer RNA (tRNA) contribute critically to its biological functions 16. In messenger RNA (mRNA), modifications often exhibit strong site specificity and ultimately influence gene expression 17. Moreover, through these mechanisms, RNA methylation modifications may also impact cellular processes such as proliferation, differentiation, and apoptosis, thereby playing important roles in the development of various diseases (Figure 1, Table 1) 18,19.

### m^2^G RNA modification

#### Introduction to m^2^G methylation

N2-methylguanosine (m^2^G) is a relatively conserved RNA modification, initially discovered in tRNAs from human cells 20. Subsequent studies have identified m^2^G modifications in certain small RNAs and mitochondrial RNAs as well 18,21.

The formation of m^2^G relies on the action of “writer” (methyltransferases). Several methyltransferases responsible for m^2^G modification have been identified in humans, including TRMT11, THUMPD2, and THUMPD3. These enzymes typically function in coordination with the cofactor protein TRMT112 (known as Trm112 in Saccharomyces cerevisiae) 18,22,23. These methyltransferases belong to Class I enzymes characterized by a Rossmann fold 18, a structural motif that enables the binding of the methyl donor S-adenosylmethionine (SAM) and stabilizes the methyltransferase structure through interaction with TRMT112 13. Moreover, cooperative action of the methyltransferase THUMPD3 and its partner protein TRMT112 enables specific recognition of the G6 position and the 3′-CCA terminus, with an intact tRNA tertiary structure being indispensable for recognition by the modifying enzyme 23. Similarly, the TRMT11-TRMT112 complex is responsible for the formation of methylguanosine at position 10 in tRNA 20,24. During this process, TRMT112 serves as a critical scaffold, enhancing the interaction between methyltransferases and tRNA and facilitating the capture of the free methyl donor SAM 13. Once methylguanosine is formed, it can induce structural alterations in rRNA subunits, subsequently influencing translation and protein synthesis. Interestingly, evidence suggests that methylation at these two specific positions may act synergistically in terms of both formation and function 18. In addition, recent studies have confirmed the presence of m^2^G modifications in certain small nuclear RNAs (snRNAs) and mitochondrial RNAs in human cells (Figure 1) 18,21.

#### Functions of m^2^G modification

The m^2^G modification exerts distinct functional effects depending on its presence in different RNA species. Studies have demonstrated that the formation and functional roles of m^2^G at positions 6/7 and 10 are mutually enhanced. Specifically, when the m^2^G modification occurs at positions 6/7 of tRNA, it reduces the accumulation of 80S ribosomal monomers, thereby promoting translation efficiency and slightly enhancing protein synthesis. In contrast, the m^2^G modification at position 10 alone has not been reliably shown to affect protein synthesis. Interestingly, when both the m^2^G modifications at positions 6/7 and 10 coexist, the translational promotion effect of m^2^G at position 6/7 is significantly amplified 18. A previous study reported that, in THUMPD3-knockout cell lines, the levels of tRNA m²G at position 6 in tRNAs bearing guanosine at this site are decreased, accompanied by a marked suppression of global protein translation and inhibition of cell proliferation 23. This suggests that the m^2^G modification at position 6 plays a positive role in regulating cell proliferation and protein synthesis. Additionally, in Thermococcus kodakarensis, the 2-amino group of guanosine at position 10 in tRNA is converted, via the intermediate m²G10, to m^2^_2_G10 by the archaeal Trm11 (tRNA m²G10/ m^2^_2_G10 methyltransferase), and this modification is thought to promote correct tRNA folding and maintain its structural stability at high temperatures 25.

Beyond tRNA, the THUMPD2-TRMT112 complex catalyzes the formation of m^2^G at position 72 of U6 small nuclear RNA (snRNA), a modification that is crucial for pre-mRNA splicing, although the precise mechanisms remain incompletely understood 18. Furthermore, in mitochondria, m^2^G modifications are generated from N2,2-dimethylguanosine (m^2^_2_G) via the action of the demethylating enzyme ALKBH7. These modifications play a key role in maintaining mitochondrial function and regulating the processing of nascent mitochondrial tRNAs 26.

In conclusion, m^2^G modifications on tRNAs are widely distributed in human cells and play vital roles in promoting global protein synthesis and cell proliferation. Moreover, rare m^2^G modifications at specific RNA sites, such as those found in snRNA and mitochondrial RNA, may participate in pre-mRNA splicing and the stabilization of RNA structures. While substantial progress has been made in understanding m^2^G modifications, the molecular mechanisms underlying this modification remain incompletely elucidated, warranting further investigation (Figure 1, Table 1).

### Cm RNA modification

#### Introduction to Cm methylation

The 2′ position is the RNA ribose position that undergoes modification in biological systems. In this modification, the hydrogen on the 2′-hydroxyl group of the ribose nucleotide is replaced by another group 27,28. Its methylation is one of the most widely occurring RNA modifications 29. This modification primarily appears in tRNA and rRNA molecules. Cm, a type of 2′-O-methylation, is formed by the methylation of the 2′-hydroxyl group of the ribose in cytidine. The Cm modification was first identified in the 1970s in Escherichia coli 30, yeast 31, and mammalian cells 32,33. Since then, this modification has been widely detected in a variety of prokaryotic and eukaryotic cells. The formation of Cm32 in t RNASer1 and t RNAGln2 in E. coli is catalysed by the enzyme TrmL 34, which is responsible for the addition of Cm34 to E. coli tRNALeu (anticodon CAA) 35. Cm32 is formed in yeast when Trm7 interacts with Trm732 36. In humans, the Cm32 and Gm34 modifications of tRNA^phe^ are catalyzed with the assistance of FTSJ1 37. In archaea, Cm is the most common modification in tRNA, and Cm56 is formed at position 56 under the catalysis of the tRNA methyltransferase Trm56 38. In prokaryotic 16S and 23S rRNA molecules, 2′-O-methylation modifications, including Cm, account for a significant proportion of the total modifications, ranging from 64% to 86% of the modified residues 28. The dual substrate rRNA methyltransferase TlyA catalyzes the formation of Cm in both 16S and 23S rRNA 39. Moreover, recent studies have shown that, similar to the m²G modification, a THUMP domain-containing tRNA methyltransferase, TrmTS, catalyzes the formation of Cm6 in Thermococcus kodakarensis tRNA^Trp^ (Figure 1) 40.

#### Functions of Cm Modification

2′-O-methylation primarily occurs through recognition on pyrimidines and plays a role in regulating gene expression, limiting the ability of nucleosides at the 2′ position to act as proton donors 41-44. In tRNA, the presence of Cm effectively prevents the hydrolysis of the phosphodiester backbone 45. In the cap structure of mRNA, Cm helps distinguish self from non-self RNA 37.

Cm modification influences the hydrogen-bonding interactions of nucleosides, thereby affecting their conformation in the RNA chain 46. Studies have shown that Cm stabilizes the C3′-endo conformation of the ribose ring compared to the C2′-endo conformation 45,47, thus stabilizing the RNA double-stranded structure 48. The thermal stability of the modified nucleic acid chain also increases 49. Notably, when Cm is present at the first position of the anticodon, it significantly enhances the efficiency of tRNA recognition of the G-end codon 50. These modifications, which alter nucleic acid properties, are likely closely related to conformational changes induced by Cm. Interestingly, when Cm is present at the second or third position of the anticodon, it may reduce codon recognition efficiency 50. Therefore, in organisms, tRNAs typically have Cm modifications at the first nucleotide of the anticodon 51-57, while such modifications are absent at the second or third positions (Figure 1, Table 1).

### m^5^U RNA modification

#### Introduction to m^5^U methylation

The m^5^U modification is widely distributed in various RNA types. It is most abundant in tRNA, typically located at position 54 in the T-loop of tRNA 58. In addition to tRNA, this modification is also found in a variety of other coding and non-coding RNAs, such as tmRNA 59, rRNA, and mRNA 60,61.

The enzymes catalyzing the formation of the m^5^U modification, as well as the m^2^G modification, belong to class I methyltransferases. These enzymes possess a Rossmann fold structure, which facilitates the transfer of a methyl group from S-adenosylmethionine (SAM) to the target nucleoside 12. Specific enzymes responsible for m^5^U modification exist in bacteria, yeast, and mammals 62-67. In human cells, the methyltransferase TRMT2A (tRNA methyltransferase 2 homolog A) primarily provides the m^5^U modification at position U54 in cytoplasmic tRNA 68. Meanwhile, TRMT2B (tRNA methyltransferase 2 homolog B) catalyzes the formation of m^5^U in mitochondrial tRNA and 12S rRNA in humans 69. TRMT2A and TRMT2B share similar gene sequences and structural composition with the Saccharomyces cerevisiae tRNA methyltransferase Trm2, and they are considered human homologs of Trm2. These enzymes have also been found to be associated with various human diseases (Figure 1) 70.

#### Functions of m^5^U modification

The m^5^U54 modification not only increases the thermal stability of tRNA 16, reduces translation error rates, but also enhances the binding affinity of the ribosomal A-site. Additionally, m^5^U plays a stabilizing role in the receptor stem-loop of Escherichia coli tmRNA 59. In summary, in lower organisms such as E. coli, m^5^U54 acts as a crucial regulator for tRNA maturation and ribosomal translocation during protein synthesis 71-73.

In eukaryotes, the m^5^U54 modification on initiator tRNA inhibits translation initiation and elongation 62,74. Recent studies have also revealed that the synergistic effect between Um54 and m^5^U54 modifications in human tRNALys3 can suppress Toll-like receptor 7 (TLR7)-dependent immune responses, thus preventing endogenous RNA from being recognized and attacked by the immune system (Figure 1, Table 1) 75.

### ms^2^i^6^A RNA modification

#### Introduction to ms^2^i^6^A methylation

The 2-methylthio-N6-isopentenyladenosine (ms^2^i^6^A) modification is a widely conserved modification found in transfer RNA (tRNA) with evolutionary significance 76. This modification primarily occurs at the 37th position of adenosine in tRNA and is involved in recognizing codons that start with uridine. The biosynthesis of ms^2^i^6^A involves multiple steps. Initially, isopentenyl groups are added to the N-6 nitrogen of adenosine, catalyzed by tRNA-dimethylallyl pyrophosphate transferase (tRNA-DMAPP), encoded by the miaA gene in Escherichia coli and the MOD5 gene in yeast, producing the precursor i^6^A 77. Subsequently, the MiaB enzyme, encoded by the TM0653 gene, catalyzes the attachment of a methylthio group (-SCH3) at the C(2) position of A37 through a reaction dependent on Fe2+, cysteine, and S-adenosylmethionine (SAM), completing the hypermodification 78. Notably, in mammalian mitochondria, CDK5 regulatory subunit-associated protein 1 (CDK5 RAP 1), a radical SAM enzyme homologous to the bacterial MiaB protein, specifically converts the A37 i^6^A modification in DNA-encoded tRNA into ms^2^i^6^A 76. This modification process is critical for mitochondrial translation and energy metabolism in mammals, and its disruption is closely associated with the pathogenesis of mitochondrial diseases (Figure 1).

#### Functions of ms^2^i^6^A modification

Previous studies have shown that the 2-methylthio-N6-isopentenyladenosine (ms^2^i^6^A) modification stabilizes the codon-anticodon interaction on the ribosome. When the first codon position A36 on the ribosome correctly pairs with U on tRNA, the ms^2^i^6^A modification helps maintain the stability of the codon-anticodon interaction. Conversely, if there is a mismatch at the first codon position, this modification may be weakened or destabilized 15. Furthermore, research has found that in the absence of the initial isopentenyl modification, there is a significant impact on the ribosome's ability to recognize nonsense codons. For instance, the translation efficiency of the UGA codon decreases by a factor of 60 79. Taken together, the ms^2^i^6^A modification plays a crucial role in the codon-anticodon interaction on the ribosome and the translation process of tRNA (Figure 1, Table 1).

## Techniques for Detecting RNA Modifications

Similar to the detection of other epigenetic modifications, the most established techniques for RNA methylation analysis are primarily based on liquid chromatography and mass spectrometry. Through continuous efforts and improvements, researchers have developed a variety of optimized detection protocols by combining chromatography with mass spectrometry. Advances in sequencing technologies, particularly the advent of high-throughput sequencing, have further accelerated transcriptome-wide studies of RNA modifications, enabling the precise localization of modified sites. Meanwhile, the emergence of deep learning techniques has provided novel approaches for the prediction and detection of RNA methylation sites, opening new avenues for research in this field (Table 2).

Liquid chromatography is one of the classical methods for RNA modification analysis. Prior to detection, RNA is hydrolyzed into its constituent components, which are then separated based on the differential interactions between the stationary phase and the mobile phase within the chromatographic column. This technique is simple to operate, offers good reproducibility, and enables precise quantitative analysis of RNA modifications; however, it has relatively weak qualitative capabilities. In the early studies of nucleosides, liquid chromatography was widely employed. As early as the 1970s, chromatography was used to separate methylated ribonucleoside products such as Am, Gm, Um, and Cm, leading to the discovery of methyltransferase activity in rat cells 32.

Mass spectrometry (MS) is a commonly used technique for the qualitative detection of RNA modifications. Based on electromagnetic principles, mass spectrometry separates substances according to their mass-to-charge ratios and identifies their chemical compositions. The combination of RNA mass spectrometry (RNA-MS) with reverse genetics approaches has led to the discovery of the m²G modification 23. In addition, the use of automated hydrogen-deuterium exchange coupled with mass spectrometry and unbiased proximity labeling methods has elucidated the interaction patterns between TRMT112 and methyltransferases 22,87. A novel method termed “Single Neuron RNA Modification Analysis by Mass Spectrometry” (SNRMA-MS) enables the detection and quantification of post-transcriptional nucleoside modifications at the single-cell level 88. Furthermore, nucleic acid isotope labeling coupled with mass spectrometry (NAIL-MS) has revealed a significant increase in Cm levels during the logarithmic phase of cell growth 89.

The combination of mass spectrometry (MS) and liquid chromatography (LC) represents a major approach for RNA modification analysis, leveraging the strengths of both techniques to achieve accurate qualitative and quantitative assessments of RNA modifications. This integrated strategy is particularly important for precise RNA modification sequencing. Liquid chromatography-mass spectrometry (LC-MS)-based methods mainly analyze RNA modifications at the nucleoside level, requiring enzymatic digestion of nucleic acid samples prior to analysis. Identification of modified nucleosides is typically achieved by comparison with authentic standards or RNA modification databases. Using reversed-phase liquid chromatography (LC)-mass spectrometry (MS) and tandem mass spectrometry (LC-MS/MS/MS) combined with stable isotope dilution techniques, significant differences in the abundance of certain modifications, including Cm, have been observed across different tissues 81. Moreover, hydrophilic interaction liquid chromatography coupled with mass spectrometry/mass spectrometry (HILIC-MS/MS) has been developed to analyze RNA modification-protein interactions and to identify Cm methylation sites 82. Cheng et al. successfully identified the m⁵U modification in mammalian mRNA and confirmed its methyl donor as S-adenosylmethionine (SAM) by employing N-cyclohexyl-N'-β-(4-methylmorpholinium) ethylcarbodiimide p-toluenesulfonate (CMCT) labeling coupled with liquid chromatography-electrospray ionization tandem mass spectrometry (LC-ESI-MS/MS) 60. Recently, a novel technique, ultra-high performance liquid chromatography-electrospray ionization-tandem mass spectrometry (UHPLC-ESI-MS/MS), has been developed. This method exhibits high sensitivity for detecting low-abundance modified nucleosides and enables their rapid and accurate quantification 83. In addition, Danijel Djukovic et al. employed high-performance liquid chromatography coupled with triple quadrupole mass spectrometry (HPLC/TQMS) and demonstrated that serum m²G metabolite levels are significantly elevated in patients with esophageal adenocarcinoma 90.

In addition to the aforementioned approaches, several other methods have been developed to detect RNA methylation modifications. Carter et al. established the 5-fluorouridine-induced catalytic crosslinking sequencing (FICC-Seq) method, which demonstrated that the m⁵U54 modification in cytoplasmic tRNA is catalyzed by TRMT2A in human cells 68. This technique, based on existing high-throughput sequencing platforms, covalently crosslinks methyltransferases to their target RNA residues. Reverse transcription of these crosslinked RNAs stalls at the crosslinking sites, enabling precise identification of modification sites. Fang et al developed a redox-activated chemical tagging sequencing technique (Reaction-Seq). This method exploits the chemoselective reaction between methylthio groups and oxaziridine moieties to achieve specific bioorthogonal labeling of ms²i⁶A without cross-reactivity toward canonical nucleosides, thereby enabling efficient enrichment of ms²i⁶A-modified RNA prior to sequencing and markedly improving detection accuracy 91. With the advancement of bioinformatics, several novel deep learning-based frameworks have been developed that can accurately predict RNA m⁵U modification sites, offering tremendous potential for applications in future biotechnological industries 84-86.

In summary, technological advancements have continuously provided more effective tools and methodologies for RNA modification detection. The integration of emerging technologies with classical experimental methods offers researchers a diversified set of strategies. Furthermore, the rapid development of bioinformatics and the emergence of new sequence-based prediction servers have significantly enhanced the efficiency and accuracy of RNA modification site detection, laying a solid foundation for future applications in medical diagnostics and therapeutics.

## The Relationship Between RNA Methylation Modifications and Disease

### m^2^G

Research has shown that the level of m^2^G is elevated in thyroid malignant tumor cells, potentially due to the role of this modification in cell proliferation. However, the underlying mechanisms remain incompletely understood 92. Studies have reported that the serum m^2^G levels in colorectal cancer (CRC) patients are significantly lower than those in healthy individuals, suggesting that m^2^G may serve as a potential serological biomarker for preliminary screening of colorectal tumors in the future 93. Additionally, it has been found that the simultaneous loss of the m^2^G-modifying methyltransferases TRMT11 and THUMPD3 affects the proliferation of colorectal cancer cells, indicating that the associated gene loci may become potential targets for controlling tumor proliferation 18. Further research has revealed that overexpression of ALKBH7 in liver cancer cells, breast cancer (BC) cells, and head and neck squamous cell carcinoma leads to an increase in m^2^G structures 94. The possible mechanism involves m^2^_2_G structures hindering base pairing, while ALKBH7 converts m^2^_2_G to m^2^G. The resulting monomethylated guanosine has a minimal impact on the base pairing process, promoting tumor cell proliferation. In lung cancer cells, depletion of THUMPD3 significantly impaired the cell's adaptability, negatively affecting key cellular processes such as proliferation and migration 95. However, the association between this phenomenon and m^2^G requires further investigation. Previous studies have shown that serum m²G levels are significantly higher in patients with esophageal adenocarcinoma than in healthy individuals, which may provide a reliable indicator for future screening of esophageal adenocarcinoma 90.

In addition to its role in tumors, m^2^G modifications have been shown to be effective in preventing the infection of certain retroviruses. The m^2^G modification may prevent reverse transcriptase and small nuclear capsid proteins from recognizing corresponding sites or may inhibit retrotranscription RNA replication in normal cells by compacting the structure of tRNATrp 96.

**Figure 2:**
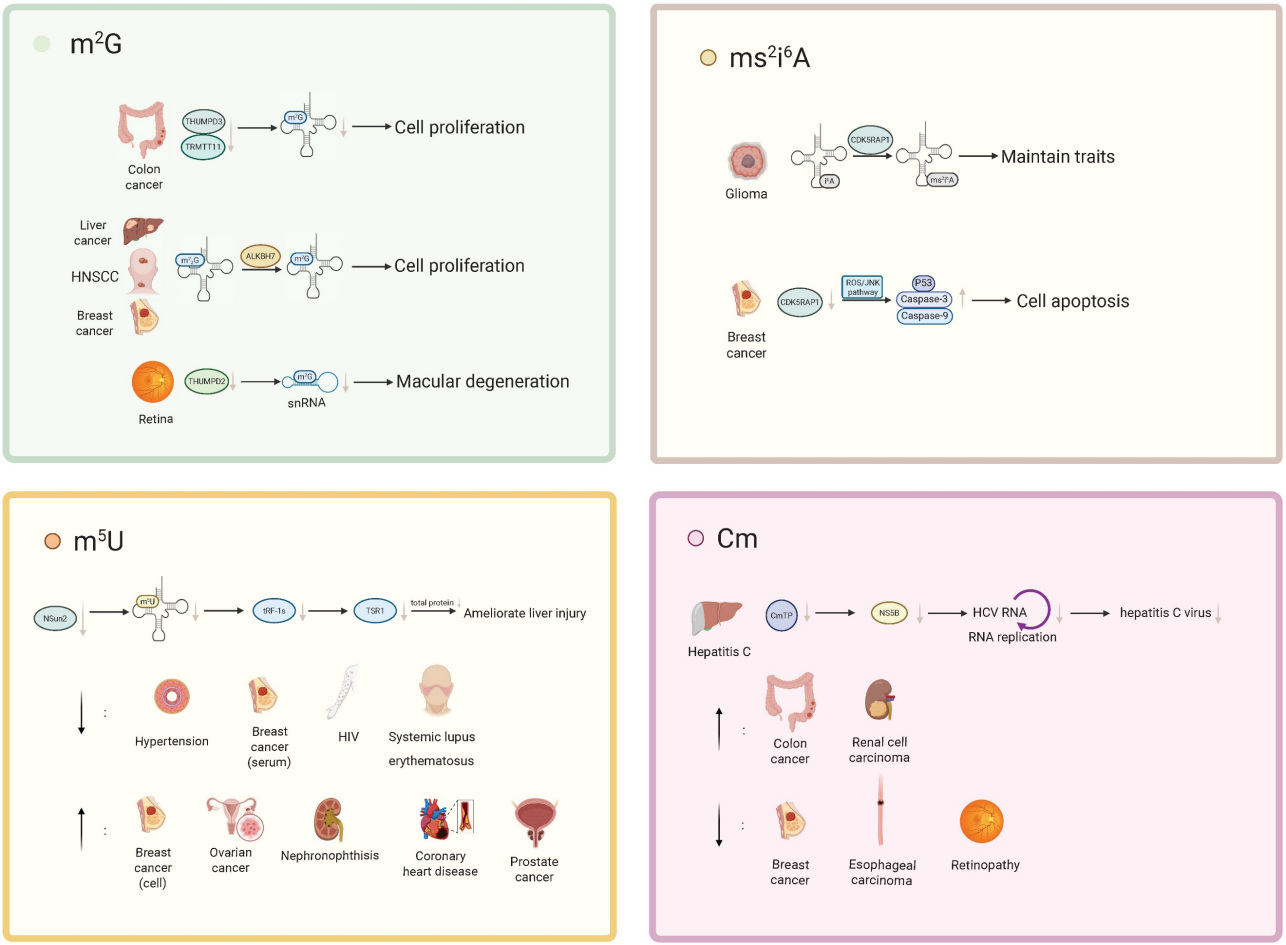
The roles of RNA m^2^G, Cm, m^5^U, and ms^2^i^6^A methylation modifications in various diseases. m²G: In colorectal cancer, concurrent loss of TRMT11 and THUMPD3 reduces m²G modification on tRNA, thereby regulating tumor cell proliferation. In hepatocellular carcinoma cells, breast cancer (BC) cells, and head and neck squamous cell carcinoma cells, ALKBH7 converts m^2^_2_G to m²G, which in turn promotes tumor cell proliferation. In the context of retinal function, loss of THUMPD2 leads to a decrease in the m²G72 modification of U6 small nuclear RNA (U6 snRNA), thereby contributing to the development of age-related macular degeneration. ms²i⁶A: CDK5RAP1 converts i⁶A to ms²i⁶A, thereby attenuating the antitumor effect of i⁶A and maintaining the stem-like properties of glioma-initiating cells (GICs). In breast cancer, CDK5RAP1 deficiency activates the ROS/JNK signaling pathway, resulting in upregulation of the p53-mediated pro-apoptotic factors caspase-9 and caspase-3 and ultimately inducing apoptosis. m⁵U: Loss of the tRNA methyltransferase NSun2 reduces tRNA m⁵C modification and diminishes the production of tRF-1s, thereby weakening their interaction with TSR1, suppressing global protein synthesis, and ameliorating liver injury. Metabolomics studies have shown that m⁵U levels are significantly decreased in patients with hypertension, in the serum of breast cancer patients, and in individuals with HIV infection or systemic lupus erythematosus, whereas m⁵U levels are markedly elevated in breast cancer cells, ovarian cancer, NPH, coronary heart disease, and prostate cancer. Cm: In hepatitis C, the triphosphate form of Cm can inhibit the activity of the hepatitis C virus (HCV) RNA polymerase NS5B, thereby suppressing HCV replication and limiting disease progression. Metabolomics analyses indicate that Cm levels are significantly increased in patients with colorectal cancer and clear cell renal cell carcinoma, but markedly decreased in patients with breast cancer, esophageal squamous cell carcinoma, and diabetic retinopathy.

A recent study indicated that THUMPD2 catalyzes the methylation of U6 snRNA m^2^G72. Knockdown of THUMPD2, which eliminates U6 snRNA m^2^G72, weakens precursor mRNA splicing activity, producing abnormally spliced precursor mRNA, which in turn leads to mRNA degradation. The loss of THUMPD2 can affect retinal function regulation and is associated with age-related macular degeneration (AMD), suggesting that abnormalities in U6 snRNA m^2^G72 modifications may contribute to the development of this disease (Figure 2, Table 3) 19.

### Cm

Metabolomic studies have identified Cm as a potential biomarker for several types of cancer, including esophageal squamous cell carcinoma 97, breast cancer 98, colorectal cancer 99,100, clear cell renal carcinoma 101, and liver cancer 102. Cm is associated with a reduced risk of esophageal squamous cell carcinoma (ESCC) and a positive correlation with colorectal cancer (CRC) risk. Serum levels of Cm are significantly lower in breast cancer patients. In clear cell renal carcinoma (ccRCC), the levels of Cm, along with other modified nucleotides and related enzymes, are elevated. Moreover, the triphosphate form of Cm can inhibit the activity of the hepatitis C virus (HCV) RNA-dependent RNA polymerase NS5B, thereby suppressing HCV replication, and this finding provides a novel therapeutic avenue for the treatment of hepatitis C 103. Recent studies have shown that Cm levels are downregulated in early diabetic retinopathy (DR), suggesting its potential as a diagnostic biomarker for DR 104. Furthermore, Cm metabolites have been found to be associated with childhood absence epilepsy (CAE) 105.

Notably, 2'-O-methylated RNA can antagonize various immune-stimulating RNAs, such as by competing with isRNAs to bind to TLR7/8, thereby inhibiting the activation of immune responses. Not only that, the modification also has the ability to activate innate immunity. This mechanism may inspire the design of drugs to modulate the immune response (Figure 2, Table 3) 106-108.

### m^5^U

Metabolomic studies on diseases such as coronary artery disease (CAD), hypertension, breast cancer, multiple sclerosis, and COVID-19 have utilized m^5^U as a metabolic biomarker for constructing disease risk assessment models 98,109-114. Notably, the upregulation of m^5^U in plasma metabolites is associated with a high risk of CAD and can serve as an assessment marker for CAD prognosis and secondary prevention 109. In contrast, m^5^U shows a negative correlation with salt-sensitive hypertension 110. Furthermore, stroke-induced depression is associated with gut metabolic dysregulation, and various metabolites, including m^5^U, are related to this phenomenon 112. Recent studies using micellar electrokinetic capillary chromatography with UV detection (MEKC-UV) to compare nucleoside levels in prostate cancer have shown that the concentrations of uridine and 5-methyluridine are elevated in cancer cells, suggesting their potential as biomarkers 115. In addition, metabolomic analyses in autoimmune diseases 113, dilated cardiomyopathy 116, and high-altitude gastric cancer 117 have also revealed alterations in m⁵U levels.

Research has shown that multiple RNA modifications, including m^5^U, are upregulated in ovarian cancer tissues. A similar phenomenon is observed in hypoxic environments, suggesting that hypoxia-associated tumor microenvironments may influence the expression of RNA modifications 118. Interestingly, *in vitro* studies on breast cancer cells have shown a significant increase in m^5^U levels in metabolic profiles 111. At the same time, a study on plasma metabolites in breast cancer patients revealed a significant decrease in m^5^U metabolites in their plasma 98, indicating that the effect of breast cancer on the generation of m^5^U metabolites remains to be further investigated.

In Nephronophthisis (NPH), the loss of Anks3 affects the metabolic pathways in the inner medullary collecting duct cells of NPH mice, resulting in m^5^U accumulation. This suggests that abnormal mutations in Anks3 may affect nucleotide metabolism by causing cellular gene damage 119.

m⁵U has been mentioned in several studies as a potential pharmacological target or therapeutic agent. 5-fluorouracil (5-FU) is a commonly used antimetabolite chemotherapeutic drug, and it has been shown that treatment of HEK293T cells with 5-FU reduces the level of 5-methyluridine in tRNA by approximately 55%, suggesting that 5-FU may exert its antitumor effect, at least in part, by perturbing normal cellular physiology through lowering m⁵U and related tRNA modifications 120. In addition, m⁵U serves as a key intermediate in the synthesis of the anti-HIV drugs stavudine (d4T) and zidovudine (AZT). Once taken up by cells, m⁵U can be phosphorylated by kinases to its mono- or triphosphate forms, which in turn inhibit viral replication. A metabolomic study in treatment-naïve individuals with HIV infection revealed a significant decrease in m⁵U levels, and demonstrated that m⁵U levels were positively correlated with CD4⁺ T cell counts, the CD4/CD8 ratio, and the proportion of CD4⁺ T cells, but negatively correlated with markers of CD8⁺ T cell activation and inflammation 121. These findings indicate that m⁵U may play a functional role in HIV therapy and is associated with anti-HIV immune activity, and that further elucidation of the mechanisms underlying m⁵U dynamics in immune cells from patients with HIV could provide additional avenues for the development of anti-HIV agents. Moreover, another study reported reduced m⁵U modification in mRNA from CD4⁺ T cells of patients with systemic lupus erythematosus 122, suggesting that m⁵U may also be implicated in the pathogenic mechanisms of immune-related diseases beyond HIV infection.

In addition, treatment of patients with chronic hepatitis B virus infection with sIFN-α significantly perturbs pyrimidine nucleoside metabolism, leading to decreased levels of 5-methyluridine, which may serve as a potential indicator for monitoring the therapeutic response to IFN-α 123. IFN-α is generally thought to exert its anti-HBV activity, at least in part, through effects on m⁶A modification 124; following IFN-α2a treatment, m⁶A modification of pgRNA is increased, thereby regulating viral RNA transcription and suppressing cDNA 125. IFN-α also induces ISG20-mediated selective degradation of m⁶A-containing HBV RNA 126. Our findings further suggest that IFN-α may additionally exert antiviral effects by altering m⁵U and other RNA modifications.

Additionally, research has demonstrated that the deletion of tRNA methyltransferase NSun2 reduces both tRNA m^5^U and m^5^C modifications while stimulating the production of stress response products, including class I tsRNAs (tRF-1s). The best mutant of tRF-1, tRF-Gln-CTG-026 (tG026), inhibits total protein synthesis by weakening the interaction between TSR1 (pre-rRNA processing protein TSR1 homolog) and tRNA, thereby improving liver injury. This mechanism provides new directions for RNA-based therapies for liver diseases 127. Interestingly, a similar mechanism is observed in the human m^5^U methyltransferase TRMT2A, where its deletion leads to low m^5^U54 modification in tRNA and the formation of tsRNAs, which are associated with cellular stress responses 128. However, the downstream signaling pathways of this mechanism and its specific relationship with RNA methylation modifications require further exploration. Notably, TRMT2A has been identified as a biomarker for increased recurrence risk in HER2+ breast cancer patients, suggesting that elucidating the physiological mechanisms related to this methyltransferase could provide breakthroughs in understanding the disease (Figure 2, Table 3) 129.

### ms^2^i^6^A

Previous studies have shown that the ms^2^i^6^A modification mediated by CDK5 regulatory subunit-associated protein 1 (Cdk5 rap 1) primarily occurs in mitochondrial DNA-encoded mt-tRNAs, and not in nuclear-encoded RNAs 76. In Cdk5 rap 1 gene knockout (KO) mice with the transverse aortic constriction (TAC) model, as well as in the stress model induced by KD, the absence of ms^2^i^6^A reduced the translation of mitochondrial DNA-encoded respiratory subunits, impairing electron transfer and oxidative respiration. This led to significant damage to heart and skeletal muscle functions due to energy metabolism insufficiency 130. Therefore, ms^2^i^6^A modification is essential for mitochondrial translation.

CDK5RAP1 deficiency induces cell cycle arrest and apoptosis in breast cancer via the ROS/JNK signaling pathway. Studies have shown that CDK5RAP1 deficiency blocks MCF-7 breast cancer cells in the G2/M phase, stimulates ROS generation, and activates p-JNK (a pro-apoptotic factor). This activation of the ROS/JNK pathway results in the overexpression of the tumor suppressor p53 and the upregulation of apoptosis factors caspase-9 and caspase-3, ultimately inducing cell apoptosis 131. In summary, CDK5RAP1 deficiency provides constructive insights for the development of novel therapies in breast cancer.

CDK5RAP1 eliminates the anti-tumor effects of i^6^A by converting i^6^A into ms^2^i^6^A, thereby protecting glioma initiating cells (GICs) from excessive autophagy triggered by i^6^A, maintaining the GIC phenotype 132. However, CDK5RAP1 is not essential for the normal conversion of i^6^A to ms^2^i^6^A in neural stem cell differentiation 132. It is understood that i^6^A inhibits GIC-associated traits and induces autophagic cell death, making targeting i^6^A a feasible approach for treating GICs (Figure 2, Table 3).

## Discussion

Recent research has highlighted the diversity and complexity of RNA modifications in cellular biology. These modifications include well-known methylations such as m^6^A, as well as other forms such as m^2^G, Cm, m^5^U, and ms^2^i^6^A methylation 20,32,58,76. Studies have revealed the physiological functions of RNA methylation modifications and their intricate connections with diseases.

Methylation modifications are crucial for the biological functions of RNA molecules. For instance, modifications like m^2^G and m^5^U in tRNA molecules alter their three-dimensional structure, affecting their role in protein synthesis 26,50,80. These modifications can influence the binding of tRNA to ribosomes, thereby impacting the efficiency and precision of protein synthesis 15,71,72,79. In mRNA, methylation modifications regulate processes such as nuclear export, splicing, degradation, and translation 18. The dynamic changes and regulation of these modifications suggest that they may play roles in disease states by influencing gene expression and function. Understanding the specific roles of these modifications in various diseases is essential for revealing the molecular mechanisms of diseases and developing new therapeutic strategies.

RNA methylation modifications have significant potential applications in cancer and other diseases. Particularly in oncology, studies have found that abnormal patterns of RNA modifications are closely associated with tumor initiation, progression, and drug resistance 94,95. m²G levels are broadly elevated in malignant thyroid tumors, hepatocellular carcinoma, breast cancer and head and neck squamous cell carcinoma, and are linked to enhanced proliferation and migration of tumor cells; loss of the related methyltransferases TRMT11 and THUMPD3 suppresses colon cancer cell proliferation, whereas ALKBH7-mediated conversion of m²_2_G to m²G promotes cell growth by improving base pairing 91-93. Serum m²G levels in patients with colorectal cancer and esophageal adenocarcinoma also differ from those in healthy individuals, suggesting that m²G and its modifying enzymes may not only participate in tumorigenic mechanisms, but may also be developed as early screening and prognostic biomarkers in liquid biopsy-based approaches 86. Cm has mainly been implicated in metabolomics studies, where its altered levels have been associated with the risk and metabolic status of esophageal squamous cell carcinoma, colorectal cancer, clear cell renal cell carcinoma and hepatocellular carcinoma; moreover, the triphosphate form of Cm can inhibit the activity of the HCV RNA polymerase NS5B, providing a potential novel target for antiviral therapy against hepatitis C 99-102.

m⁵U appears to play an even more prominent role in cancer, cardiovascular and immune-related conditions. On the one hand, elevated plasma m⁵U is associated with a higher risk of coronary artery disease and poor prognosis, and has repeatedly emerged as a key metabolite in various disease risk prediction models; on the other hand, imbalances in m⁵U and its related metabolites have been observed in hypertension, post-stroke depression, multiple sclerosis and autoimmune disorders, suggesting that m⁵U participates in the fine-tuning of cardiovascular, neurological and immune regulation 97,108-110. m⁵U also constitutes an important structural scaffold for several antitumor and anti-HIV nucleoside analogs, and its levels are closely correlated with the degree of immune reconstitution and inflammatory status in individuals with HIV infection 120. Deficiency of m⁵U methyltransferases such as NSun2 and TRMT2A can induce tsRNA production and alter stress responses, and has been linked to the attenuation of liver injury and to recurrence risk in HER2⁺ breast cancer, further underscoring the potential role of m⁵U in disease pathogenesis 110,122.

ms²i⁶A predominantly occurs in mitochondrial tRNAs and is closely related to energy metabolism. Cdk5RAP1 deficiency leads to reduced ms²i⁶A levels, impairing the translation of mitochondrial respiratory chain subunits and resulting in myocardial and skeletal muscle dysfunction, highlighting its importance in metabolic and cardiovascular diseases 129. In cancer, Cdk5RAP1 deficiency can, on the one hand, induce cell cycle arrest and apoptosis in breast cancer cells via the ROS/JNK pathway 130, while on the other hand, in gliomas it converts the antitumor modification i⁶A into ms²i⁶A to protect tumor-initiating cells 131, suggesting that ms²i⁶A may exert bidirectional, cell type and context dependent regulatory effects.

In addition, the development of RNA modification detection technologies has provided powerful tools for RNA modification research. Mass spectrometry is now capable of identifying and quantifying single modification sites, while high-throughput sequencing technologies can analyze modification patterns across the transcriptome 68. Machine learning-based site prediction methods are also being updated continuously 84-86. These technological advancements not only deepen our understanding of the biological functions of methylation and other modifications but also aid in identifying RNA modification patterns associated with diseases. With the continuous optimization of detection technologies, we expect that future studies will enable RNA modification analysis at the single-cell level, revealing new aspects of cellular heterogeneity in diseases 88.

Overall, current studies indicate that RNA methylation modifications such as m²G, Cm, m⁵U, and ms²i⁶A are not only closely associated with the occurrence, progression, and therapeutic response of various cancers, but also participate in the development and progression of multiple non-neoplastic diseases, including age-related macular degeneration, diabetic retinopathy, epilepsy, viral hepatitis, HIV infection, and autoimmune disorders. These relatively rare RNA methylation modifications and their associated regulatory enzymes therefore hold promise as novel biomarkers for disease diagnosis and prognosis, and may provide potential targets for therapeutic intervention. However, the molecular mechanisms underlying these RNA modifications remain insufficiently understood. The regulatory system of “writers-readers-erasers” has not yet been fully elucidated, and most studies are still limited to metabolite-level observations or correlative analyses. In particular, the precise modification sites, downstream signaling pathways, tissue and cell-type specificity, as well as the synergistic or antagonistic interactions among different RNA modifications remain largely unexplored. Future studies are therefore needed to systematically elucidate the roles of these RNA modifications in gene expression regulation and cell fate determination at the mechanistic level. In addition, integrating high-sensitivity quantitative detection techniques with single-cell sequencing and spatial multi-omics approaches will facilitate a deeper understanding of the dynamic changes of RNA modifications across different cell types and disease microenvironments. Meanwhile, the application of programmable RNA modification editing tools and *in vivo* disease models will help clarify the functional roles of these modifications in disease development and progression, and evaluate their feasibility and safety as diagnostic biomarkers or potential therapeutic targets in real clinical settings, thereby promoting the translation of RNA modification research toward precision medicine and clinical applications.

## Conclusion

This review focuses on four relatively rare RNA methylation marks, m²G, Cm, m⁵U and ms²i⁶A, summarizing their fundamental roles in maintaining RNA structural stability and regulating translation and gene expression, and systematically outlining current evidence linking these modifications to cancer and non-malignant diseases. Available data indicate that these marks are not only involved in the initiation, progression and treatment response of multiple malignancies, but are also closely associated with cardiovascular, metabolic and neurological disorders, as well as viral infections and autoimmune diseases, thereby highlighting their potential as fluid- or tissue-based biomarkers and therapeutic targets. However, compared with classical modifications such as m⁶A, studies on the “writer-reader-eraser” enzyme systems and the precise mechanisms of m²G, Cm, m⁵U and ms²i⁶A remain at an early stage, and most conclusions are still descriptive or correlative in nature. Future work should investigate these modifications in greater mechanistic detail and in larger, well-characterized clinical cohorts to better substantiate their translational potential in precision diagnosis and individualized therapy for cancer and other diseases.

## Figures and Tables

**Table 1:** Functions of RNA modifications

Modification	Process	Enzymes involved	Description	PMID
**m^2^G**	Protein synthesis	THUMPD3	Promotes global protein synthesis by reducing rRNA80s monomer accumulation and thereby affecting translation	18
	Cell proliferation		Cell proliferation is suppressed in THUMPD3-deficient cells.	18
	mRNA translation		Global protein translation is significantly suppressed in THUMPD3 knockout cell lines.	23
	tRNA stability	TRMT11	Promoting thermal stabilization of tRNAs at the tRNA tenth position	80
	Formation and functioning of m^2^G6/7		N. A	18
	Splicing of precursor mRNA	THUMPD2	The m^2^G structure formed at position 72 of U6snRNA is functional for precursor mRNA splicing	18,19
	Mitochondrial activity	ALKBH7	Conversion of m^2^_2_G to m^2^G on mitochondrial tRNA stabilizes mitochondrial activity and function properly	26
	Processing of nascent tRNAs in mitochondria		Reduces polycis-trans mitochondrial RNA and promotes mitochondria-encoded tRNA processing	26
**Cm**	Moderated translation	N. A	Maintaining the accuracy of tRNA recognition reading frames	41-44
	tRNA stability	TrmL,TrmJ, Trm7, aTrm56, etc	Helps to correctly identify codons and improve the heat resistance of tRNA molecules.	37,45
	mRNA stability	N. A	Cm in the cap structure of mRNA distinguishes between autologous and non-autologous RNAs.	37
	RNA stability	N. A	Altered hydrogen-bonding interactions of nucleosides stabilize RNA double strands and increase the thermal stability of nucleic acid chains	45,46,48
	Moderated translation	N. A	The anticodon first and second and third positions have opposite roles in translation efficiency	50
**m^5^U**	tRNA stability	TrmA	Involved in tRNA tertiary structure interactions and enhances tRNA tertiary structure thermal stability	16
	Moderated translation		Reduced translation errors and increased ribosomal A-site binding	59
	tmRNA stability		Stabilizing effect on the alanine structure of the stem branch of the Escherichia coli tmRNA receptor	59
	Moderated translation	Trm2	The m^5^U54 modification of tRNA promoters has an inhibitory effect on translation initiation and elongation m^5^U54 promoting tRNA modification and regulating the rate of ribosomal translocation	62,73, 74
	Immune escape	TRMT2A	Synergistic effect of Um54 and m^5^U54 modifications in tRNAs to avoid recognition and attack of endogenous RNAs by the autoimmune system	75
**ms^2^i^6^A**	mRNA translation	tRNA-DMAPP/MiaB	The ms^2^i^6^A modification of tRNA stabilizes codon-anticodon interactions on the ribosome	15
	Mitochondrial gene expression	CDK 5 RAP 1	Cdk5 Rap 1-mediated ms^2^i^6^A modification in mt-tRNA regulates mitochondrial translation and energy metabolism	15,76

Abbreviation: m^2^G, N2-methylguanosine; Cm, 2′-O-methylcytidine; m^5^U, 5-methyluridine; ms^2^i^6^A, 2-methylthio-N6-isopentenyladenosine; tRNA, transfer RNA; rRNA, ribosomal RNA; mRNA, messenger RNA; N. A, not available

**Table 2:** Detection Methods for RNA m^2^G, Cm, m^5^U, and ms^2^i^6^A modifications.

Methods	Description	Advantages	Limitations	PMID
Chromatography	It is a physicochemical separation method that utilizes properties such as solubility and adsorption of substances.	Convenient operation, high sensitivity, accurate quantitative results, can separate complex multi-component mixtures.	Difficulty in completing qualitative analysis. Conventional chromatography takes a long time to detect.	32
RNA-MS	Mass spectra are obtained by focusing ions with different mass-to-charge ratios at different points in a magnetic field to determine their mass.	Qualitative analysis can be completed with accurate results.	Not suitable for analysis of complex compounds.	23
LC-MS	Combining chromatography and mass spectrometry.	Combining the advantages of chromatography and mass spectrometry.	The operation process is complex and costly.	83-85
LC-MS/MS	Combining chromatography and mass spectrometry.	It is more efficient compared to LC-MS, with less sample dosage, faster analyzing speed, and wider detection range.	The operation process is complex and costly.	83-85
Reversed-phase liquid chromatography	Liquid chromatography in which the polarity of the mobile phase is greater than the polarity of the stationary phase.	High efficiency, high separation capacity, and universal applicability.	Polar and hydrophilic compounds are rarely or not retained.	83
HILIC	The stationary phase of the HILIC column is hydrophilic, and the more hydrophilic the compound, the longer it is retained.	Suitable for separation of strongly polar and hydrophilic compounds.	Not suitable for separating non-polar compounds, and the separation efficiency of the column deteriorates after a period of use.	84
ESI-MS	Electrospray is utilized to generate ions that apply a high voltage to a liquid to create an aerosol.	Overcomes the tendency of macromolecules to break up when ionized.	Sensitivity is affected by spray voltage.	63
UHPLC	Liquid chromatography using small-particle, high-performance particulate stationary phases.	Increases analytical throughput, sensitivity and peak capacity.	Instrument maintenance costs are high.	85
FICC-Seq	Modification sites were determined using covalent cross-linking principles and high-throughput sequencing.	Efficient and Accurate.	High Costs.	71
Reaction-Seq	ms²i⁶A was specifically bioorthogonally labeled via chemoselective reactions.	allows detection of ms²i⁶A modification at single-nucleotide resolution	complex and costly.	87
Machine learning models	Modification sites were predicted by computerized processing of the data.	Simple operation, high efficiency and low cost.	Possible errors and accuracy could be improved.	88-90

Abbreviation: RNA-MS, RNA-mass spectrometry; LC-MS, liquid chromatography-mass spectrometry; LC-MS/MS, liquid chromatography-tandem mass spectrometry; HILIC, hydrophilic interaction liquid chromatography; ESI-MS, electrospray ionization-mass spectrometry; UHPLC, ultrahigh performance liquid chromatography; FICC-Seq, Fluorouracil Induced-Catalytic-Crosslinking-Sequencing.

**Table 3:** Specific role of modifications in diseases

Modification	Disease	Enzyme	Target	Description	PMID
m^2^G	CRC	THUMPD3	Position 6 of tRNA	Reduced expression of THUMPD3, THUMPD2, and TRMT11 inhibits global protein synthesis and thus cell proliferation	18
		TRMT11	Position 10 of tRNA		
	Liver cancer	ALKBH7	mtRNA	Transformation of mt-RNA m^2^_2_G to m^2^G may unblock base pairing by m^2^_2_G, thereby promoting tumor cell proliferation	93
	BC				
	HNSCC				
	Lung cancer	THUMPD3	N. A	Depletion of THUMPD3 significantly impairs cellular adaptability and negatively affects cell proliferation and migration.	94
	AMD	THUMPD2	snRNA	Deletion of THUMPD2 eliminates the U6 m2G72 on the 'spliceosome', resulting in aberrant splicing of pre-messenger RNAs, associated with AMD	19
	EAS	N. A	N. A	The serum level of m²G is significantly higher in patients with esophageal adenocarcinoma than in healthy controls.	86
Cm	BC	N. A	N. A	Reduced serum Cm levels in breast cancer patients	97
	CRC	N. A	N. A	Elevated Cm positively correlates with colorectal cancer (CRC) risk	98-99
	ccRCC	N. A	N. A	Increased Cm levels in clear cell renal carcinoma (ccRCC)	100
	ESCC	N. A	N. A	Elevated Cm is associated with reduced risk of esophageal squamous cell carcinoma (ESCC)	96
	Hepatitis C	N. A	N. A	The triphosphate form of Cm can inhibit the activity of the hepatitis C virus (HCV) RNA polymerase NS5B, thereby suppressing HCV replication.	102
	DR	N. A	N. A	Cm levels are downregulated in DR	103
m^5^U	OC	N. A	N. A	m^5^U expression level is up-regulated in ovarian cancer tissues, possibly due to hypoxia-associated tumor microenvironment affecting m^5^U expression	117
	BC	N. A	N. A	m5U was significantly elevated in serum from breast cancer patients and reduced in breast cancer cells cultured *in vitro*	97,110
	NPH	N. A	N. A	Deletion of Anks3 affects inner medullary collecting duct cell metabolism in NPH mice, showing accumulation of m5U	118
	Hypertension	N. A	N. A	m5U urinary metabolites are inversely associated with hypertension	109
	CAD	N. A	N. A	increased plasma levels of m5U in CAD patients at high risk of major clinical outcomes	108
	Prostate cancer	N. A	N. A	The concentration of m⁵U is elevated in prostate cancer and may serve as a potential biomarker.	114
	HIV	N. A	N. A	m⁵U levels are positively correlated with CD4⁺ T cell counts, the CD4/CD8 ratio, and the proportion of CD4⁺ T cells.	120
	Liver disease	NSun2	N. A	NSun2 deficiency ameliorates liver injury by reducing the production of tRF-1s, thereby weakening TSR1 interactions.	126
ms^2^i^6^A	BC	CDK5RAP1	ROS/JNK pathway	CDK5RAP1 deficiency induces human breast cancer MCF-7 cell cycle arrest and apoptosis through the ROS/JNK signaling pathway	130
	Glioma	CDK5RAP1	i^6^A	Conversion of i^6^A to ms^2^i^6^A abolishes the antitumor effect of i^6^A and protects GIC from excessive autophagy triggered by i^6^A and maintains GIC traits	131

Abbreviation: m^2^G, N2-methylguanosine; Cm, 2′-O-methylcytidine; m^5^U, 5-methyluridine; ms^2^i^6^A, 2-methylthio-N6-isopentenyladenosine; CRC, colon cancer; BC, breast cancer; HNSCC, head and neck squamous cell carcinoma; AMD, macular degeneration; EAS, esophageal adenocarcinoma; ccRCC, clear cell renal cell carcinoma; ESCC, esophageal squamous cell carcinoma; DR, diabetic retinopathy; OC, ovarian cancer; NPH, nephronophthisis; Anks3, ankyrin and sterile alpha motif (SAM)-containing 3; CAD, coronary artery disease; tRNA, transfer RNA; rRNA, ribosomal RNA; mRNA, messenger RNA; N.A, not available

## Data Availability

The authors confirm that all data supporting the findings of this study are available in the main manuscript.
